# Plasma microRNAs, miR-223, miR-21 and miR-218, as Novel Potential Biomarkers for Gastric Cancer Detection

**DOI:** 10.1371/journal.pone.0041629

**Published:** 2012-07-30

**Authors:** Bo-sheng Li, Yong-liang Zhao, Gang Guo, Wei Li, En-dong Zhu, Xiao Luo, Xu-hu Mao, Quan-ming Zou, Pei-wu Yu, Qian-fei Zuo, Na Li, Bin Tang, Kai-yun Liu, Bin Xiao

**Affiliations:** 1 Clinical Microbiology and Immunology, College of Medical Laboratory Science, Third Military Medical University, Chongqing, China; 2 General Surgery and Center of Minimally Invasive Gastrointestinal Surgery, Southwest Hospital, Third Military Medical University, Chongqing, China; 3 Pharmacy, Southwest Hospital, Third Military Medical University, Chongqing, China; Baylor University Medical Center, United States of America

## Abstract

**Background:**

MicroRNAs (miRNAs), endogenous small non-coding RNAs, are stably detected in human plasma. Early diagnosis of gastric cancer (GC) is very important to improve the therapy effect and prolong the survival of patients. We aimed to identify whether four miRNAs (miR-223, miR-21, miR-218 and miR-25) closely associated with the tumorigenesis or metastasis of GC can serve as novel potential biomarkers for GC detection.

**Methodology:**

We initially measured the plasma levels of the four miRNAs in 10 GC patients and 10 healthy control subjects by quantitative reverse transcription polymerase chain reaction (qRT-PCR), and then compared plasma miRNA results with the expressions in cancer tissues from eight GC patients. Finally, the presence of miR-223, miR-21 and miR-218 in the plasma was validated in 60 GC patients and 60 healthy control subjects, and the areas under the receiver operating characteristic (ROC) curves of these miRNAs were analyzed.

**Results:**

We found that the plasma levels of miR-223 (*P*<0.001) and miR-21 (*P*<0.001) were significantly higher in GC patients than in healthy controls, while miR-218 (*P*<0.001) was significantly lower. The ROC analyses yielded the AUC values of 0.9089 for miR-223, 0.7944 for miR-21 and 0.7432 for miR-218, and combined ROC analysis revealed the highest AUC value of 0.9531 in discriminating GC patients from healthy controls. Moreover, the plasma levels of miR-223 (*P*<0.001) and miR-21 (*P* = 0.003) were significantly higher in GC patients with stage I than in healthy controls. Furthermore, the plasma levels of miR-223 were significantly higher in GC patients with *helicobacter pylori* (Hp) infection than those without (*P* = 0.014), and significantly higher in healthy control subjects with Hp infection than those without (*P* = 0.016).

**Conclusions:**

Plasma miR-223, miR-21 and miR-218 are novel potential biomarkers for GC detection.

## Introduction

Gastric cancer is the fourth most common malignancy in the world and the second leading cause of cancer death in both sexes worldwide. The highest mortality rates are estimated in Eastern Asia [1]. The 5-year survival rate for gastric cancer is less than 20%–25% in the USA, Europe and China [2]. Currently, complete surgical resection is the most effective treatment, offering an excellent (90%) chance of a cure for patients with early gastric cancer [3]. For advanced gastric cancer, despite curative surgery, about 80% of the patients die within a short time from locoregional recurrence (87%) and/or distant metastasis (30%) [4]. Therefore, improvement in early diagnosis could increase the chance of a cure in early GC patients or prolong the survival of patients with early-stage GC. However, most early-stage GCs are difficult to detect [5]. The conventional serum markers for GC, such as carbohydrate antigen 19-9 (CA19-9) and carcinoembryonic antigen (CEA), lack sufficient sensitivity and specificity to facilitate early detection.

MicroRNAs (miRNAs) are small, non-coding RNAs that posttranscriptionally regulate gene expression. Aberrant expression of miRNAs has been correlated with several diseases, including cancers [6]. Previous studies have indicated that tissue miRNA expression profiles can be considered as diagnostic biomarkers in cancer [7]–[9]. However, this diagnostic method is limited in its efficacy as tissue specimens are not convenient to access and are invasive to obtain. An increasing number of papers are reporting that circulating miRNAs are stably detected in various body fluids, including serum and plasma [10], [11]. Circulating miRNAs as novel stable biomarkers would be one of the most promising means of diagnosis, because plasma and serum are easy to access and noninvasive to obtain. Recently, several promising serum or plasma miRNA biomarkers for GC detection have been identified [12], [13].

To explore that novel plasma miRNA signatures can distinguish patients with GC (particularly early-stage GC) from healthy controls, we selected four miRNAs (miR-223, miR-218, miR-25 and miR-21) which had been reported to be frequently dysregulated in GC tissue and closely correlated with tumorigenesis or metastasis of GC [14]–[21]. We supposed that the plasma levels of the three miRNAs (miR-223, miR-218 and miR-25) were aberrant in GC patients as well as those of miR-21, which suggested that this signature can serve as a biomarker for GC detection [12]. However, the plasma levels of miR-21 in GC patients at different TNM stages have not been identified. In this study, we compared the plasma levels of the four miRNAs in GC patients to healthy controls, and evaluated the feasibility of the four miRNAs as novel noninvasive biomarkers for GC detection.

## Materials and Methods

### Patients and Samples

All samples were collected from consenting individuals according to the protocols approved by the Ethics Review Board at Third Military Medical University. In total, 70 patients with GC prior to any treatments and 70 healthy control subjects from Southwest Hospital (Chongqing, China) were included in our study between 2010 and 2011. For the 70 GC patients, we analyzed the histology of the GC tissues, including 56 Adenocarcinoma, 13 Mucinous adenocarcinoma and 1 Signet-ring cell carcinoma, and determined the tumor locations, including 34 in Gastric body, 24 in Gastric antrum, 8 in Gastric cardia and 4 in others (Upper stomach, Gastric angle, Gastric stump). The GC patients were classified according to the clinical TNM stages, including 12 stage I (median age, 53 years [range, 36–70 years]; 8 male, 4 female), 11 stage II (median age, 61 years [range, 36–77 years]; 7 male, 4 female), 36 stage III (median age, 55 years [range, 30–71 years]; 26 male, 10 female), and 11 stage IV (median age, 53 years [range, 46–66 years]; 9 male, 2 female).The status of Hp infection were tested using Anti-HP Antibodies ELISA Diagnostic Kits (S20010005), (BEIJING BEIER BIOENGINEERING CO., LTD), showing 43 GC patients with Hp infection and 27 without. For the 70 healthy control subjects, 44 male and 26 female were included, and the median age was 51 years [range, 26–75 years]. The results of Hp infection test showed 31 healthy control subjects with Hp infection and 39 without. (Table 1).

**Table 1:** Clinical feature of the gastric cancer (GC) patients and healthy control subjects in the training set and the validation set.

Virable	Gastric cancer N = 70 (%)	Normal control N = 70 (%)	*P*-value
**Gender**			0.371
Male	49 (70)	44 (63)	
Female	21 (30)	26 (37)	
**Age**			0.398
Median (range)	54 (30–77)	51(26–75)	
≥55	36 (51)	31 (44)	
<55	34 (49)	39 (56)	
***Helicobacter pylori*** ** status**			**0.042**
Infection	43 (61)	31 (44)	
Non-infection	27 (39)	39 (56)	
**Tumor location**			
Body	34 (49)		
Antrum	24 (34)		
Cardia	8 (11)		
Other	4 (6)		
**Histology**			
Adenocarcinoma	56 (80)		
Mucinous adenocarcinoma	13 (19)		
Signet ring cell cancer	1 (1)		
**TNM stage**			
I	12 (17)		
II	11 (16)		
III	36 (51)		
IV	11 (16)		
**Lymph node status**			
Metastasis	52 (74)		
No metastasis	18 (26)		

Note: *P* values are from two-sided χ^2^ test.

Cell-free plasma was isolated from all blood samples within 2 hr of collection using a two-step protocol (1,500 r.p.m. for 10 min, 12,000 r.p.m. for 2 min) to prevent contamination by cellular nucleic acids. Plasma was transferred to a fresh tube, leaving a fixed height of 0.5 cm plasma supernatant above the pellet to avoid disturbing the pellet [11]. It was stored at −80°C in 450 µl aliquots. Eight pairs of tissue samples were collected from eight GC patients with higher levels of plasma miR-223 and miR-21, and lower levels of plasma miR-218 than healthy controls after surgical resection, and approximately cut to 1 mm squares and immediately frozen in liquid nitrogen.

### RNA Extraction

Total RNAs were extracted from 400 µl of plasma using the mirVana PARIS Kit (Ambion) according to the manufacturer’s protocol, and eluted with 105 µl of pre-heated (95°C) Elution solution. To allow for the normalization of sample-to-sample variation in the RNA isolation step, 10 µl of 0.05 µM synthetic *C. elegans* miR-39 (synthetic RNA oligo-nucleotides synthesized by GenePharma) was added to each denatured sample after combining the plasma sample with Denaturing Solution [11]. For the frozen tissues, total RNA was extracted using TRIzol reagent (Invitrogen) according to the manufacturer’s protocol, and finally resuspended in 60 µl of pre-heated (95°C) nuclease-free water.

### Quantitative Reverse-transcriptase Polymerase Chain Reaction (qRT-PCR)

The reverse transcription reaction was carried out using a Taqman MicroRNA Reverse Transcription Kit (Applied Biosystems). cDNA was synthesized in 5 µl volumes containing 1.67 µl of RNA extract, 0.5µl of 10×reverse transcription buffer, 0.05 µl of 100 mM dNTPs, 0.063 µl of RNase Inhibitor (20 U µl^−1^), 0.33 µl of Mutiscribe Reverse Transcriptase (50 U µl^−1^), 0.5 µl of gene-specific primer and 1.887 µl of nuclease-free water. The reactions were incubated at 16°C for 30 min, followed by 42°C for 30 min, then 85°C for 5 min before being held at 4°C. The synthesized cDNA was diluted 2 fold by nuclear-free water. Quantitative PCR reactions were carried out using 2 µl of cDNA solution, 5 µl of TaqMan 2×Perfect Master Mix (Takara), 0.25 µl of gene-specific primers/probe (TaqMan® MicroRNA Assays, Applied Biosystems. Table S1) and 2.75 µl of nuclease-free water in a final volume of 10 µl, and run on a Bio-Rad IQ5 (Bio-Rad Laboratories, Inc) thermocycler. The reaction mixtures were incubated at 95°C for 2 min, followed by 40 cycles of 95°C for 15 s and 60°C for 30 s. The cycle threshold (*C*_t_) values were calculated with the Bio-Rad iQ5 2.1 Standard Edition Optical System Software 2.1.94.0617.

The plasma miRNA concentrations were calculated using a standard curve constructed using synthetic miRNAs [22]. The standard reference miRNAs were amplified for each reaction. However, the expression of miRNAs from tissue samples was normalized using the 2^−ΔΔ^ *C*_t_ method from the *C*_t_ values of the miRNAs of interest relative to RNU6B.

### Normalization of Experimental qRT-PCR Data from Plasma using Synthetic C. elegans miR-39

*C. elegans* miR-39, which lacks sequence homology to human miRNAs, was selected to normalize the experimental qRT-PCR data. Known quantities of synthetic *C. elegans* miR-39 were diluted to produce *C*_t_ values within the *C*_t_ value ranges of the miRNA standard curves. We empirically added 10 µl of 0.05 µM synthetic *C. elegans* miR-39 to 400 µl of plasma after combining the plasma sample with Denaturing Solution. The *C. elegans* miR-39 was amplified as well as other miRNAs. The following formula was used for adjusting the *C*_t_ values of miRNAs (miR-223, miR-21, miR-218 and miR-25) in all plasma samples: Normalized_ *C*_t_ value for the miRNA in the sample  =  Raw_ *C*_t_ value − [(SpikeIn_ Average_ *C*_t_ value of the given sample) − (Median_ SpikeIn_*C*_t_)] [11]. The normalized_ *C*_t_ value was then used to calculate the concentration of each miRNA.

### Statistical Analysis

The Mann-Whitney test was used to compare the differences in plasma miRNA concentrations and the miRNA ratios between the cancer group and the healthy group. A two-sided χ^2^ test was used to compare the differences in gender, age or Hp infection status between the GC patients and the healthy controls. ANOVA test was used to analyze the relationship between the levels of miR-223, miR-21, miR-218 and TNM stages. Receiver-operating characteristic (ROC) curves and the area under the ROC curve (AUC) were used to assess the feasibility of using plasma levels of miRNAs as diagnostic tools for detecting GC. The Youden index was used to select the optimal cutoff values. A *P* value of less than 0.05 was considered statistically significant. All statistical analyses were performed using SPSS 13.0 software and graphs were generated using GraphPad Prism 5.0 (Graphpad Software Inc, Caligornia).

## Results

### Characterizes of Subjects

One hundred and forty subjects, including 70 GC patients and 70 healthy control subjects, were recruited into this study. No significant differences in gender or age were found between the GC patients and the healthy controls (*P* = 0.371, *P* = 0.398, χ^2^ test, respectively). Hp infection status was significantly different between the GC patients and the healthy controls (*P* = 0.042) (table 1).

### Evaluation of Quantitative RT-PCR for Measuring the miRNAs in Plasma

**Figure 1:**
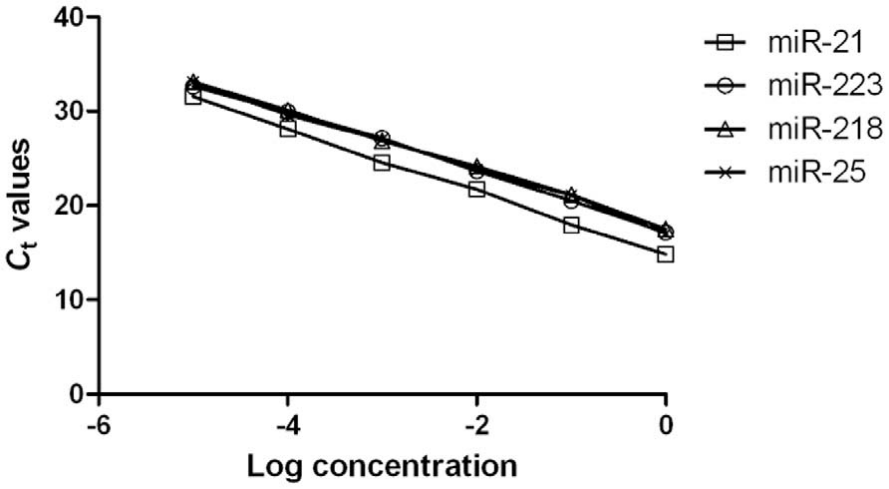
Standard curve of miR-21, miR-223, miR-218 and miR-25 using synthetic miRNAs. Ten-fold serial dilution of synthetic miRNA was used to generate the standard curves. Linearity was confirmed within these concentrations for miR-21, miR-223, miR-218 and miR-25 ranging from 0.1 to 0.000001 fmol. (*miR-21*: y = −3.337x+14.77 (R^2^ = 0.999); *miR-223*: y = −3.121x+17.37 (R^2^ = 0.997); *miR-218*: y = −3.071x+17.79 (R^2^ = 0.998); *miR-25*:y = −3.114x+17.47 (R^2^ = 0.993)).

To define the dynamic range and sensitivity of miRNA quantification by real-time PCR, the synthetic single-strand miRNAs were serially diluted 10-fold from concentrations of 0.1 to 0.000001 fmol for miR-223, miR-218, miR-25, and miR-21 on the recommendation of the GenePharma miRNA Reference Panel. The linearity of the quantitative RT-PCR between the logarithmic values of the input miRNAs and the *C*_t_ values was confirmed for each synthetic miRNA, miR-223, miR-218, miR-25 and miR-21 (R^2^ = 0.997, R^2^ = 0.998, R^2^ = 0.993 and R^2^ = 0.999, respectively) (Figure 1).

### Preliminary Screening of Plasma miRNAs which can Monitor GC

**Figure 2:**
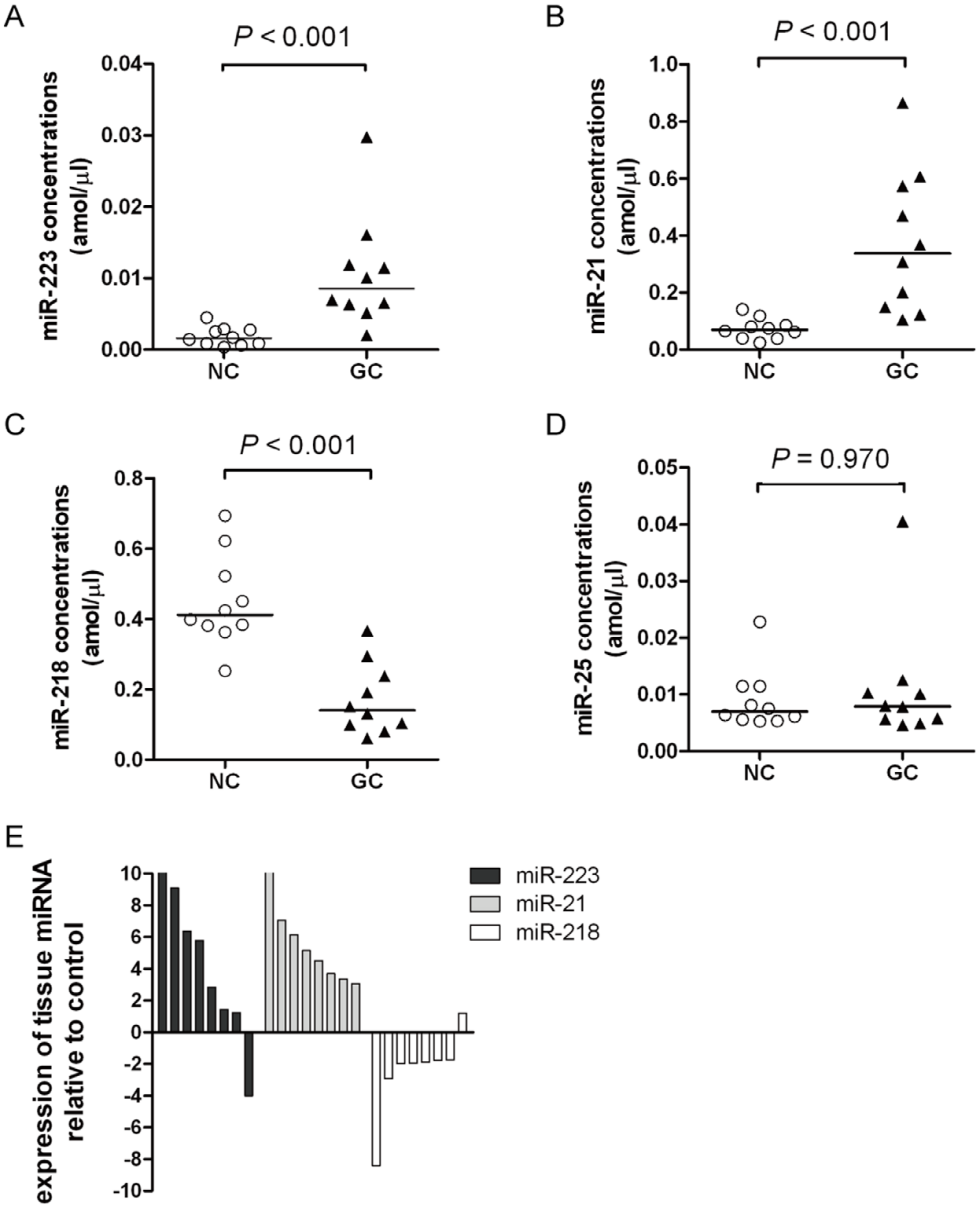
The expression profile signatures of four miRNAs in the initial analysis. Scatter dot plots of the plasma levels of four miRNAs in gastric cancer patients (GC, n = 10) and healthy control subjects (NC, n = 10). Scatter dot plots show the plasma levels of *miR-223* **(A**), *miR-21* **(B)**, *miR-218* **(C)** and *miR-25* **(D)**. The lines in the scatter dot plots denote the medians. (**E**) Bars of the expression levels of miR-223, miR-21 and miR-218 in primary GC tissues and paired normal tissues. The expression levels of miR-223 were higher in primary GC tissues in seven of the eight patients analyzed (87.5%) and miR-21 in eight patients (100%), whereas miR-218 was lower in seven patients (87.5%) than in paired normal tissues. All assays were repeated three times in duplicates.

Four miRNAs, miR-223, miR-218, miR-25 and miR-21, were aberrantly expressed in GC tissues. To investigate whether the levels of the four miRNAs present dysregulation in plasma of patients with GC, we initially measured the plasma levels of the four miRNAs in 10 GC patients and 10 healthy controls. As expected, the plasma levels of miR-223 and miR-21 were significantly higher in GC patients than in healthy controls (*P*<0.001), whereas miR-218 were significantly lower (*P*<0.001). However, the plasma levels of miR-25 were not significantly different between the GC patients and the healthy controls (*P* = 0.970) (Figure 2(A-D)). It has been reported that the levels of miR-21 in GC plasma could reflect those in primary GC tissue [12]. To investigate whether the levels of three miRNAs (miR-223, miR-218 and miR-21) in GC plasma can reflect those in primary GC tissue, we tested the levels of the three miRNAs in eight pairs of GC tissue and adjacent normal tissue samples from the 8 GC patients whose plasma levels of miR-223, miR-21 were significantly higher, and miR-218 was significantly lower. As shown in Figure 2E, the expression levels of miR-223 were higher in primary GC tissues than in controls in seven of the eight patients analyzed (87.5%) and miR-21 in eight patients (100%), whereas miR-218 was lower in seven patients (87.5%), suggesting that the levels of these three miRNAs in GC plasma reflected those in primary GC tissue.

### The Plasma Levels of miR-223, miR-21, miR-218 and miR-25 were Validated in Large Scale

**Figure 3:**
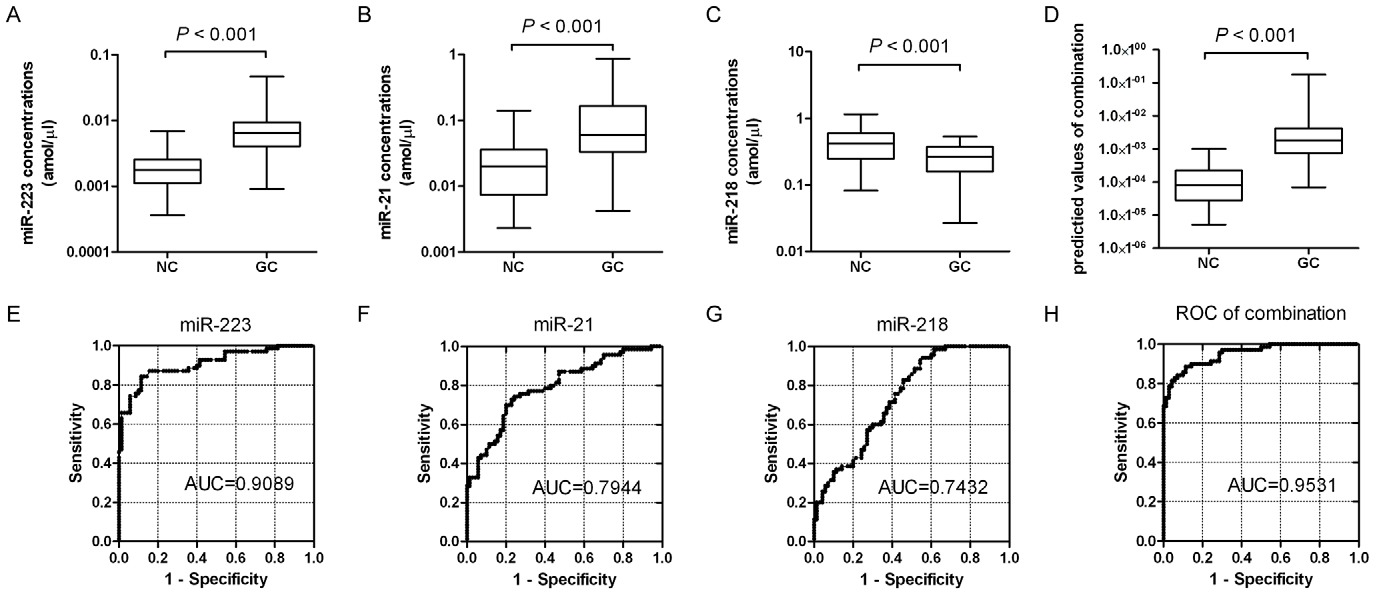
Validation of the plasma levels of miR-223, miR-21 and miR-218 in large scale. Box plots show the plasma concentrations of miR-223 **(A)**, miR-21 **(B)**, miR-218 **(C)** and (miR-223×miR-21)/miR-218 **(D)** in GC patients (GC, n = 70) and healthy controls (NC, n = 70). The lines inside the boxes denote the medians. The whiskers of box plots: Min to Max. Graphs of receiver operating characteristics (ROC) curve show the area under the curves (AUCs) of miR-223 **(E)**, miR-21 **(F)**, miR-218 **(G)** and (miR-223×miR-21)/miR-218 **(H)** for distinguishing the GC patients from the healthy controls. The interval between the 5^th^ and 95^th^ percentiles denotes the confidence level and the report results show as Fraction. All assays were repeated three times in duplicates.

To evaluate the plasma levels of above four miRNAs as diagnostic markers for GC detection, another 60 GC patients and 60 healthy control subjects were added in this validation assay. As shown in Figure 3, the levels of miR-223 and miR-21 were significantly higher in GC plasma than in control (*P*<0.001), while miR-218 was significantly lower (*P*<0.001). However, the plasma levels of miR-25 were not significantly different between the GC patients and the healthy controls (*P* = 0.082) (Figure S1). The concentration values of the four miRNAs measured in the plasma of subjects were shown in Table S2. ROC curve analyses were performed to evaluate the diagnostic value for the three plasma miRNAs and revealed that the three plasma miRNAs were valuable biomarkers for distinguishing GC from normal controls with AUCs (areas under the ROC curve) of 0.9089 (95% CI: 0.8598 to 0.9580) for miR-223, 0.7944 (95% CI: 0.7211 to 0.8677) for miR-21 and 0.7432 (95% CI: 0.6628 to 0.8236) for miR-218. At the optimal cutoff value of 0.7286 with the value of sensitivity + specificity-1 considered to be maximal for miR-223, the sensitivity and specificity were 84.29% and 88.57%; at the cutoff value of 0.5000 for miR-21, the sensitivity and specificity were 74.29% and 75.71%, and at the cutoff of 0.3858 for miR-218, the sensitivity and specificity were 94.29% and 44.29%. To elevate the diagnosis value, the combination ROC curve analyses were performed by (miR-223 multiplied by miR-21) divided by miR-218. The ratio of (miR-223×miR-21)/miR-218 yielded the highest AUC value of 0.9531 (95% CI: 0.9222 to 0.9839) and the optimal cutoff value of 0.7715, the sensitivity and specificity were 84.29% and 92.86%, which indicated that the combination signature has a strong potential diagnosis value for GC detection.

### The Plasma Levels of miR-223, miR-218 and miR-21 in GC Patients with different Clinical Status

**Figure 4:**
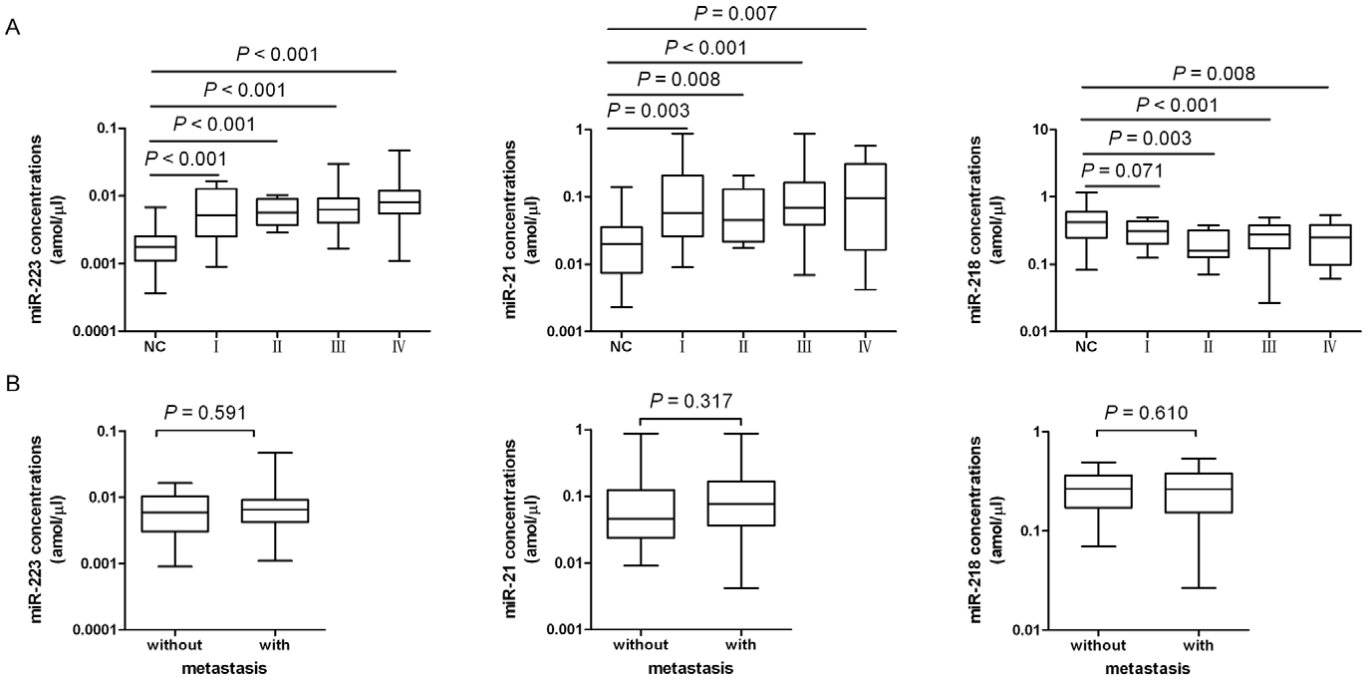
Evaluation of the plasma concentrations of miR-223, miR-21 and miR-218 in GC patients at different clinical status. (A) Box plots of the plasma concentrations of miR-223 (left panel), miR-21 (middle panel) and miR-218 (right panel) in healthy controls (NC, n = 70) and gastric cancer (GC, n = 70) patients at different TNM stages (12 with I, 11 with II, 36 with III and 11 with IV). (B) Box plots of the plasma concentrations of miR-223 (left panel), miR-21 (middle panel) and miR-218 (right panel) in GC patients with or without metastasis. The lines inside the boxes denote the medians. The whiskers of box plots: Min to Max.

The plasma levels of these three miRNAs in the GC patients at different TNM stages (12 with I, 11 with II, 36 with III or 11 with IV) were analyzed to determine whether the three plasma miRNAs can detect early-stage GC. As shown in Figure 4A, the plasma levels of the three miRNAs were not significantly different across four stages (miR-223, *P* = 0.244; miR-218, *P* = 0.664; miR-21, *P* = 0.596), however, each of the four stages including stage I patients had significantly elevated plasma miR-223 and miR-21 when compared with the healthy controls (P<0.01), and miR-218 was significantly decreased in stage II, III and IV (*P*<0.01), whereas miR-218 were not significantly different between the stage I patients and the healthy controls (*P* = 0.071). Furthermore, we compared the levels of the three miRNAs in plasma from the GC patients with metastasis to those without. As shown in Figure 4B, the plasma levels of the three miRNAs had no significant differences between the GC patients with metastasis and those without (miR-223, *P* = 0.320; miR-218, *P* = 0.979; miR-21, *P* = 0.310).

### Relationship between the Plasma Levels of miR-223, miR-21, miR-218 and Hp Infection Status of Subjects

**Figure 5:**
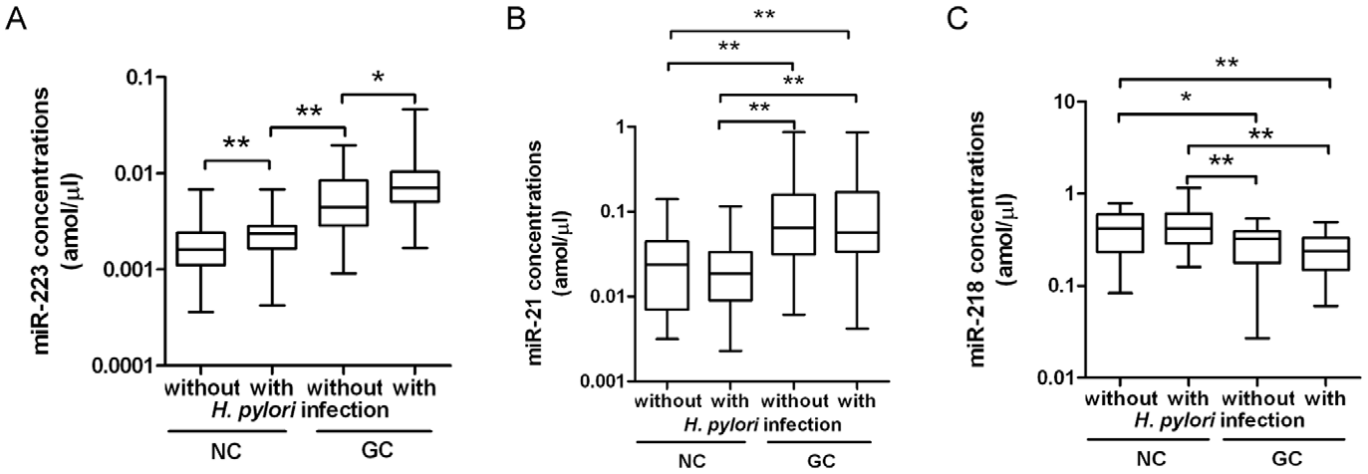
Analyses of the plasma concentrations of miR-223, miR-21 and miR-218 in subjects with H. *pylori* infection and those without. Box plots of the plasma concentrations of miR-223, miR-21 and miR-218 in GC patients with *H. pylori* infection (With, n = 43) and those without (Without, n = 27), and in healthy control subjects with *H. pylori* infection (With, n = 31) and those without (Without, n = 39). Note: *, *P*<0.05; **, *P*<0.01. Box plots show the plasma levels of miR-223 (A), miR-21 (B) and miR-218 (C). The lines inside the boxes denote the medians. The whiskers of box plots: Min to Max.

In addition, we tested the status of Hp infection in the 140 subjects as described above, and analyzed the plasma levels of the three miRNAs in the 70 GC patients (43 with Hp infection and 27 without) and the 70 healthy control subjects (31 with Hp infection and 39 without) to evaluate the relationship between the plasma levels of the three miRNAs and Hp infection status of the subjects. As shown in Figure 5, the plasma levels of miR-21 (*P* = 0.875, *P* = 0.527) and miR-218 (*P* = 0.097, *P* = 0.539) were not significantly different between the GC patients with Hp infection and those without, and between the healthy controls with Hp infection and those without, respectively. However, miR-223 was significantly higher in the GC patients with Hp infection than those without (*P* = 0.014), and significantly higher in the healthy controls with Hp infection than those without (*P* = 0.016). The plasma levels of miR-223 (*P*<0.001) and miR-21 (*P*<0.001) were significantly elevated in the GC patients with Hp infection or whose without when compared with the healthy controls with Hp infection or whose without, whereas miR-218 was significantly lower (*P*<0.05). These data provide strong evidences that the three miRNA signatures can distinguish GC patients with or without Hp infection from healthy controls.

## Discussion

In this study, the levels of four miRNAs (miR-223, miR-218, miR-21 and miR-25) in plasma from 70 GC patients and 70 healthy controls were analyzed. Consistent with the previous studies by Tsujiura et al [12], our assay also showed that the significantly higher plasma levels of miR-21 in GC patients. We found that the levels of miR-223 were significantly higher in GC plasma than in control, whereas miR-218 was significantly lower. The combined ROC analyses revealed a highest AUC of 0.9531 with 84.29% sensitivity and 92.86% specificity for the ratio of (miR-223×miR-21)/miR-218 in discriminating GC from controls. We also analyzed the plasma levels of miR-223, miR-21, miR-218 in GC patients at different clinical status (TNM stage or metastasis) and the relationship between the plasma levels of the three miRNAs and Hp infection status of the subjects.

Although miR-223 has been reported to be nearly exclusively expressed in bone marrow [23], its overexpression has been observed in many types of cancer, such as esophageal carcinoma [16], hepatocellular carcinoma [24], and GC [14], [15]. Recently, Xiaohua Li reported that miR-223 was only overexpressed in metastatic gastric cancer cells and stimulated non-metastatic gastric cancer cells migration and invasion [25]. Why the plasma levels of miR-223 were significantly higher in patients with early-stage GC? In the GC microenvironment, many tumor-associated cells, such as macrophages, myeloid cells, dendritic cells and T cells, have the capacity to release exosomes, which shuttle both mRNA and microRNA to other cells or circulation [26]. For early-stage GC, miR-223 might be up-regulated in some tumor-associated cells and delivered into the peripheral blood via exosmes. Recent evidence indicated that miR-223 released by macrophages was shuttled into breast cancer cells and regulated the invasiveness of breast cancer cells [27]. It has been demonstrated that the restoration of miR-218 suppresses Robo1 expression and inhibits gastric cancer cell invasion and metastasis *in vitro* and *in vivo* [17]. Overexpression of miR-218 resulted in a significantly decreased cell growth activity and cell invasion of AGS cells compared with that of the control [20]. Gao C et al [21] reported that the expression levels of miR-218 were reduced significantly in GC tissues, in H. pylori-infected gastric mucosa, and in H. pylori-infected AGS cells. In our study, the plasma levels of miR-218 were not significantly different between GC patients with Hp infection and those without, or between healthy control subjects with Hp infection and those without. The levels of miRNAs might be different between GC plasma and gastric mucosa. MiR-21 is overexpressed in various cancers, including breast cancer [28], lung cancer [29], colon cancer [30], and GC [18], [19]. Although Tsujiura et al [12] reported that the plasma levels of miR-21 were significantly elevated in GC patients, its levels in plasma from GC patients at different TNM stags have not been identified.

Increasing number of papers reported that circulating miRNAs can serve as noninvasive biomarkers for GC detection. Recently, Hanshao Liu reported that serum miR-378 was significantly elevated in the GC patients with TNM stage I, suggesting that this miRNA signature can serve as a novel noninvasive biomarker for early detection of GC [31]. But the Hp infection status in the GC patients and healthy controls were not mentioned. It is well known that Hp infection is one of the major causes of GC, including gastric adenocarcinoma, gastric MALT lymphoma. If the plasma/serum levels of the specific circulating miRNAs were only dysregulated in GC patients with Hp infection but not in those without, the miRNAs might serve as biomarkers for the detection of patients with Hp infection instead of the detection of patients with GC.

In this study, although we analyzed the plasma levels of miR-223, miR-21 and miR-218 in GC patients at different TNM stages, the number of early-stage GC samples was modest. The number of plasma miRNAs tested in training set was limited. In the future investigation, we may access more number of early-stage GC samples to evaluate the role of plasma miR-223, miR-21, miR-218 or other plasma miRNAs associated with GC in early detection of GC.

For the purpose of searching effective blood-based biomarkers for GC detection to prolong the survival of patients with early GC, many researchers have focused on circulating miRNAs, which have recently been reported to serve as an effective and non-invasive biomarker for detecting various cancers or other diseases [32]–[35]. Although the sensitivity and specificity of circulating miRNA biomarkers for GC detection are much higher than that of the serum biomarkers (CA19-9 and CEA) currently used, it is a long way to go before circulating miRNAs as a clinical diagnosis are used to detect GC, because the levels of a circulating miRNA might be significantly higher or lower in various diseases. Future studies of circulating miRNA biomarkers may focus on combining the expression profiles of circulating miRNAs from all common diseases to obtain the specific biomarkers for unique disease detection. Although Jianning Song [36] recommended miR-16 and miR-93 as suitable reference genes for serum miRNA analysis for GC patients and healthy controls, the sample size is modest. The normalization methods used to determine the levels of circulating miRNAs should be unified.

In conclusion, we identified that three plasma miRNAs (miR-223, miR-21 and miR-218) can potentially serve as novel noninvasive biomarkers for GC detection. Whether miR-223 and miR-21 have a capability to detect early-stage GC will be identified in future studies.

## Supporting Information

Figure S1 **Validation of the plasma levels of miR-25 in GC patients and healthy controls.** Box plots of the plasma concentrations of miR-25 in GC patients (GC, n = 70) and healthy controls (NC, n = 70). The lines inside the boxes denote the medians. The whiskers of box plots: Min to Max. No significant difference was observed between GC patients and healthy control subjects.
(TIF)

Table S1 **The mature microRNAs and their matched primer/probe AB assay ID.**
(DOCX)

Table S2 **The concentration values of the four miRNAs measured in the plasma of subjects.**
(XLSX)
