# Supplementary figures and images for: Plasma microRNAs, miR-223, miR-21 and miR-218, as Novel Potential Biomarkers for Gastric Cancer Detection
Source: PLoS One. 2012 Jul 30;7(7):e41629. doi: 10.1371/journal.pone.0041629 (PMC3408505; doi:10.1371/journal.pone.0041629)

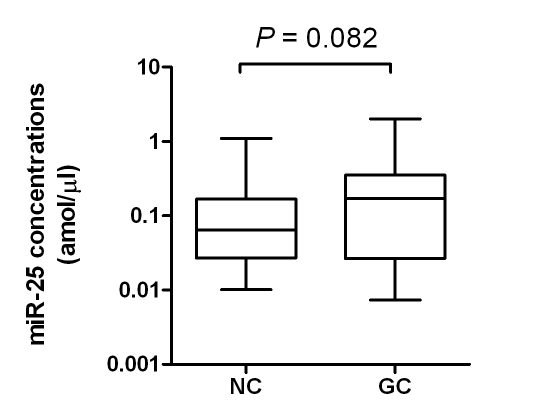

Supplement: Figure S1 — Validation of the plasma levels of miR-25 in GC patients and healthy controls. Box plots of the plasma concentrations of miR-25 in GC patients (GC, n = 70) and healthy controls (NC, n = 70). The lines inside the boxes denote the medians. The whiskers of box plots: Min to Max. No significant difference was observed between GC patients and healthy control subjects. (TIF) [file pone.0041629.s001.tif]
