# Supplementary material for: Plasma microRNAs, miR-223, miR-21 and miR-218, as Novel Potential Biomarkers for Gastric Cancer Detection
Source: PLoS One. 2012 Jul 30;7(7):e41629. doi: 10.1371/journal.pone.0041629 (PMC3408505; doi:10.1371/journal.pone.0041629)
Supplement: Table S1 — The mature microRNAs and their matched primer/probe AB assay ID. (DOCX) [file pone.0041629.s002.docx]

| **microRNA** | **Mature Sequence** | **Primer/probe AB assay ID** |
| --- | --- | --- |
| Hsa-miR-223 | UGUCAGUUUGUCAAAUACCCCA | 002295 |
| Hsa-miR-21 | UAGCUUAUCAGACUGAUGUUGA | 000397 |
| Hsa-miR-218 | UUGUGCUUGAUCUAACCAUGU | 000521 |
| Hsa-miR-25 | CAUUGCACUUGUCUCGGUCUGA | 000403 |
| Cel-miR-39 | UCACCGGGUGUAAAUCAGCUUG | 000200 |
